# Announcemen: EuAs C_2_S 4th Eurasia Conference on Chemical Sciences

**DOI:** 10.1155/MBD.1994.340

**Published:** 1994


					Vol. 1, No. 4, 1994 Announcements
EuAsC2S
4th EURASIA CONFERENCE
ON CHEMICAL SCIENCES
including New Developments in Inorganic/Physical/Theoretical, Environmental
and Medicinal Chemistry
Kuala Lumpur, Malaysia
December 17 21, 1994
Hosted by Institut Kimia Malaisia with the cooperation of the Federation of Asian Chemical Societies
and of the Federation of European Chemical Societies
Introduction
The Eurasia Conference on Chemical Sciences was first held in Bangkok, Thailand in 1988 to bring
together scientists from the supercontinent of Eurasia for an improventent of mutual relations as
well as an enhancement of scientific cooperation in the field of chemical research. The success has
resulted in similar conferences being held in 1990 (Seoul) and 1992 (Bangkok).
Location and Venue
The 4th Eurasia Conference on Chemical Sciences will be held from 17 to 21 December 1994 at
the Federal Hotel in Kuala Lumpur, Malaysia.
It is hoped that, as previously, a large number of distinguished chemists not only from the
supercontinent but also from other continents will participate.
Scientific Programme
The Conference will comprise plenary lectures and mini-symposia (including one one
"Metal-Based Drugs"). Prominent chemists will be invited to present Keynote Addresses at the
Mini-symposia.
Contributory papers are also welcomed in all branches of chemical sciences, especially in newly
emerging areas, as well as those of special significance to the developing countries in the region,
such as:
Bioinorganic Chemistry
Environmental Chemistry
Polymer Chemistry
Organometalli Chemistry
Solution Chemistry
Catalysis
Agrochemistry
Colloid and Surface Chemistry
Computational Chemistry
Natural Products Chemistry
Solid State Chemistry
Synthetic Chemistry
Chemistry of New Materials
Industrial and New Process Chemistry
Macromolecular Chemistry
English will be the official language of the Conference. A Chemical and Industrial
Exposition"CHEMINDEX EURASIA '94" will be held in conjunction with the Conference.
All correspondence should be addressed to
Professor Hitoshi Ohtaki
Department of Chemistry
Faculty of Science and Engineering
Ritsumeikan University
1916 Noji-cho, Kusatsu
Shiga 525, Japan
Fax: + 81 775 61 26 59
Mr Lim Teck Thai, Hon. Secretary
Institut Kimia Malaysia
129B, Jalan Aminuddin Baki
Taman Tun Dr. Ismail
60000 Kuala Lumpur, Malaysia
Tel./Fax: + 603 718 99 09
+ 603 756 7511
Intending contributors are invited to send titles of their papers and
abstracts (not exceeding one page, A4 size) by May 1, 1994.
Registration fees
Before September 30, 1994: US $ 200 (accompanying persons: US $100)
After September 30, 1994: US $ 250 (accompanying persons: US $130)
340

				

